# HIV‐related stigma, couple relationship quality, and mental health in sero‐discordant pregnant couples in Kenya

**DOI:** 10.1111/aphw.70120

**Published:** 2026-01-28

**Authors:** Asuman Buyukcan‐Tetik, Turan Deniz Ergun, Bulent Turan, Reshmi Mukerji, Kevin Owuor, Abigail Hatcher, Elizabeth A. Bukusi, Zachary Kwena, Anna Helova, Evelyne Owengah, Lynae Darbes, Janet M. Turan

**Affiliations:** ^1^ Utrecht University Utrecht the Netherlands; ^2^ Sabanci University Istanbul Turkey; ^3^ Department of Psychology, Health, and Technology, Faculty of Behavioural Management and Social Sciences University of Twente Enschede the Netherlands; ^4^ Department of Psychology Koc University Istanbul Turkey; ^5^ School of Public Health University of Alabama at Birmingham Birmingham USA; ^6^ University of North Carolina at Chapel Hill Chapel Hill North Carolina USA; ^7^ Kenya Medical Research Institute Nairobi Kenya; ^8^ Department of Health Behavior and Clinical Sciences, School of Nursing University of Michigan Ann Arbor Michigan USA

**Keywords:** African region, couple relationships, HIV, mental health, stigma

## Abstract

HIV‐related stigma negatively impacts the health of people who are living with HIV. Stigma may also affect sero‐discordant couples where one partner is living with HIV, but the other is not. However, we know little about how HIV‐related stigma and couple relationship quality jointly affect depression and anxiety in both the individual and their partner. We analyzed dyadic data from 491 sero‐discordant pregnant couples in southwestern Kenya collected during 2019–2022 using Actor‐Partner Interdependence Model (APIM) methods. Controlling for relationship quality, HIV‐related stigma perceived by both women and men was detrimental to their own mental health as well as to their partner's mental health. High relationship quality was associated with better mental health of couple members, independent of stigma, but reporting high relationship quality did not significantly buffer the negative effect that stigma had on mental health. The partner effects of women's and men's relationship quality were sometimes in opposite directions: women's reports of higher relationship quality were negatively associated with men's depressive symptoms; however, men's reports of higher relationship quality were positively associated with higher depressive symptoms in women. These results suggest that interventions should support sero‐discordant couples to resist and reduce HIV‐related stigma as well as build positive couple relationships.

## INTRODUCTION

Human Immunodeficiency Virus (HIV) remains a serious health problem. Approximately 40 million people are living with HIV, and 630,000 died of AIDS‐related causes in 2023 worldwide (UNAIDS, 2024). In sub‐Saharan Africa, where the epidemic continues to be concentrated, 62% of all new infections are among women and girls (UNAIDS, 2024). HIV‐related stigma has been extensively investigated (Mahajan et al., 2008; Relf et al., 2021; Yigit et al., 2024) and continues to be a major public health and human rights concern. HIV‐related stigma has been conceptualized as a process that reproduces social difference and, in doing so, “feeds upon, strengthens, and reproduces existing inequalities of class, race, gender, and sexuality” (Parker & Aggleton, 2003). As pre‐existing gender inequalities may feed into the HIV stigmatization process, it is expected that HIV stigma may have particularly harmful effects on women's mental and HIV‐related health outcomes.

Manifestations of HIV‐related stigma include social isolation and exclusion for people living with HIV (PLWH) (Dlamini et al., 2007; Mahajan et al., 2008). The gendered nature of HIV‐related stigma means that it can be particularly damaging for women, and marriage, abandonment, and physical, sexual, and emotional violence are some of the more commonly reported manifestations (Mukerji et al., 2023). Male partners may be the perpetrators of this violence and can reinforce internalized stigma through verbal abuse (Marais et al., 2019), which adds to women's emotional distress (Simbayi et al., 2007). However, HIV‐related stigma may also impact the partner not living with HIV based on perceptions of stigma and attitudes about HIV in society. Therefore, it is crucial to understand whether and how HIV‐related stigma impacts the couple dyad: both the actor (PLWH) and the partner (relationship partner not living with HIV).

Stigma experiences are often associated with mental health symptoms such as depression and anxiety for PLWH. A large body of literature has reported on the association of HIV‐related stigma with depression, anxiety, emotional distress, and post‐traumatic stress symptoms (Brandt, 2009; MacLean & Wetherall, 2021; Rueda et al., 2016; Simbayi et al., 2007; Waldron et al., 2021; Yigit et al., 2024). Turan, Hatcher, et al. (2017) described how HIV‐related stigma can lead to poor HIV outcomes through compromised psychological resources, poor mental health, and stress processes. An important stigma dimension is perceptions of stigma in the community. Perceived stigma may make it more likely that PLWH internalize this stigma and anticipate stigma experiences, resulting in adverse psychosocial and health outcomes (Turan, Budhwani, et al., 2017).

While it has been found that women living with HIV are more likely to have worse overall mental health compared to men living with HIV (MacLean & Wetherall, 2021; Orza et al., 2015; Waldron et al., 2021), what is less well known is whether HIV‐related stigma affects the mental health of both partners in HIV‐affected couples. The intimate nature of couple relationships suggests that experiences of stigma and discrimination may act as a stressor for both partners, even if only one partner has directly experienced discrimination (Wofford et al., 2019). For example, studies with Chinese heterosexual sero‐discordant couples have suggested that HIV stigma leads to mental distress among partners (Huang et al., 2018; Yu et al., 2016).

While HIV‐related stigma may lead to mental distress for couples living with HIV, the quality of the couple relationship may potentially increase resilience and mitigate some of the harms caused by HIV‐related stigma (Huang et al., 2018). It has been posited that couples employing a communal coping style, where the relationship goals are prioritized over self‐oriented goals, are better able to cope with HIV‐related stigma (Rogers et al., 2016). Relationship quality indicators such as dyadic adjustment, commitment, intimacy, and communication have been shown to be associated with lower HIV risk behaviors (Ruark et al., 2018). Couples with higher sexual satisfaction and partner social support have reported lower levels of anticipated stigma, while negative communication styles were associated with greater anticipated stigma (Gutin et al., 2023). Better relationship quality, mediated by “love attitudes” (feelings of being in love, committed, and emotionally attached), may reduce self‐stigma and improve mental health (Yang et al., 2017). However, there is still a need to understand if such positive relationship dynamics among couples can reduce the effects of stigma and promote mental health for couples affected by HIV.

This study employs a theoretical framework based on the Interdependence Model of Communal Coping and Behavior Change for couples (Lewis et al., 2006). This framework suggests that relationship factors can spur behavior change and improve health outcomes, and there is evidence for this in Kenyan couples (Musoke et al., 2022; Rogers et al., 2016). Lewis's Interdependence Model suggests three principal components through which this occurs (Lewis et al., 2006). Predisposing factors (perception of health threat, preferences for outcomes, relationship functioning, communication style, gender) lead to spouses' transformation of motivation (interpreting health threat as significant for partner and relationship, and thus, responding accordingly), which then leads to couples' communal coping (using strategies such as couple communication, joint decision‐making and planning, and working together to engage in positive health behaviors).

These components may ultimately result in health‐enhancing behaviors and improved health outcomes for the couple. This process could potentially translate to improved mental health and well‐being for both partners within the dyad (Leach et al., 2013; Sarno et al., 2022). This theoretical framework has been successfully applied to HIV prevention strategies and HIV‐related behaviors in sub‐Saharan African settings (Darbes et al., 2019; Montgomery et al., 2012; Rogers et al., 2016) and elsewhere (Starks et al., 2018).

Despite such research, gaps remain in understanding whether both HIV‐related stigma and relationship quality, as experienced by each member of the couple, affect mental health for both partners in HIV sero‐discordant relationships. This paper seeks to investigate this issue. Moreover, we aimed to examine whether couple relationship quality moderates the effect of HIV‐related stigma on mental health in HIV‐status discordant married couples in Kenya. This information on psychosocial pathways that affect mental health in sero‐discordant couples is essential for the design of programs to support the health and well‐being of couples affected by HIV.

## METHOD

### Setting

The Kenyan counties included in this study (Migori and Kisumu) have some of the highest HIV prevalence rates in the country, with 13–17% of adults 15–64 years of age testing HIV‐positive (National AIDS and STI Control Programme (NASCOP), 2022). This setting is a priority area for HIV prevention efforts addressing pregnant women and male partners due to the high HIV prevalence among pregnant women and continuing high rates of mother‐to‐child transmission of HIV (7–8%) (NASCOP, 2018). Rates of marriage, either formal or informal, and co‐habitation of marital partners are high in this setting. Polygyny, in which men may have more than one wife, is still practiced in some communities in the region.

### Study design

Data for the current analyses are derived from the baseline assessments of a randomized trial of interventions for pregnant couples (the Jamii Bora Study) conducted in Migori and Kisumu Counties during the period 2019–2022 (Kwena et al., 2021). Pregnant women (both living and not living with HIV) were recruited from antenatal clinics of 24 different government health facilities in these counties, with the following eligibility criteria: maximum of 36 weeks of pregnancy, 15 years of age or older, had been offered HIV testing at a participating antenatal care clinic, was currently in a stable relationship (for at least six months) with a male partner and living with that male partner, and had not yet participated in a couple HIV testing and counseling (CHTC) during this pregnancy. By design, pregnant women living with HIV were over‐sampled (2/3 of the sample at baseline) to have a sufficient sample size for examining HIV outcomes such as maternal viral load and HIV‐free infant survival.

If the woman was eligible and interested, a researcher obtained informed consent for study participation, conducted the baseline questionnaire, and obtained permission to contact her male partner. With the woman's permission, the team subsequently contacted her primary male partner, arranged to meet with him, obtained informed consent, and conducted his baseline questionnaire separately. Due to the primary outcome of the intervention trial being uptake of CHTC and facilitated disclosure, mutually disclosed HIV‐positive concordant couples were not recruited for this study. Data for the current analyses are from these baseline questionnaires conducted before any study intervention activities.

### Data collection and management

Baseline questionnaires were interviewer‐administered on Android tablets programmed using the Open Data Kit (ODK) Collect software in the participant's preferred language (Swahili, Luo, or English). Data were checked by the local Study Coordinator and uploaded regularly to a secure server at the University of Alabama at Birmingham. Baseline questionnaires were completed by a total of 967 women and 800 male partners, with a total of 800 couples recruited and both completing baseline questionnaires. Of these 800 couples, in 491 the woman had HIV (confirmed from medical records) and her male partner did not (self‐report), in 253 neither partner had HIV, in 12 the man had HIV and his female partner did not, in 10 both partners had HIV at baseline, and in 34 one of the couple members had unknown HIV status. For the current analyses, we analyzed data from the 491 HIV sero‐discordant couples in which the woman was living with HIV according to her clinic record, and the man self‐reported not living with HIV. All participants provided individual written informed consent and were reimbursed 500 Kenyan shillings [KSh] (roughly 5 US dollars) for their time/travel related to participating in the questionnaire. The study was approved by the ethics committees of the Kenya Medical Research Institute and the University of Alabama at Birmingham, and is registered with clinicaltrials.gov [#NCT03547739]. Information about the sociodemographic characteristics of the sample is provided in Table S1 in the Supplemental Materials.

### Measures

The baseline questionnaires included questions and validated scales (translated into local languages and used previously in this Kenyan setting) related to HIV testing behaviors, couple relationship dynamics, stigma, mental health, and healthcare utilization. Each member of the couple completed the questionnaire separately. The following measures were utilized in the current analyses: all variables were computed using the averages of the related items.

#### Mental health

*Depression* was measured using an 8‐item self‐reported measure of depressive symptoms (PHQ8 scale; Kroenke et al., 2009). This has Likert scales with scores ranging from 0 (*not at all*) to 3 (*nearly every day*) for symptoms present within the past two weeks. Higher scores corresponded to higher depressive symptoms. Cronbach's alpha in the current sample was .89.

*Anxiety* was measured using the 7‐item Generalized Anxiety Disorder Scale (GAD7 scale; Spitzer et al., 2006). This self‐reported measure of anxiety severity assesses the frequency of symptoms ranging from 0 (*not at all*) to 3 (*nearly every day*) within the past two weeks. Higher scores represent higher anxiety symptoms. Cronbach's alpha in the current sample was .89.

#### Stigma

The variable *negative attitudes about people living with HIV* (referred to as negative attitudes in this paper) were measured by a scale developed by Genberg et al. (2009). This sub‐scale of a more comprehensive community‐level HIV‐related stigma scale measures negative attitudes towards PLWH. This 5‐point Likert scale measures perceptions about how PLWH are treated in the community, such as whether they face verbal abuse, ejection from their homes, or neglect from their families (for the complete list of items, see Table S2 in the Supplemental Materials). Higher scores corresponded to more negative attitudes about PLWH. Cronbach's alpha in the current sample was .88.

The scale assessing *perceived community stigma against people living with HIV* (referred to as perceived stigma in this paper) was also developed by Genberg et al. (2009). This additional sub‐scale of the more comprehensive community‐level HIV‐related stigma scale measures perceptions of stigma and discrimination faced by PLWH in the community. This 5‐point Likert scale measures negative attitudes and beliefs about PLWH, such as blame for infection, whether they should be ashamed for having HIV or be isolated for having HIV, and negative feelings such as PLWH are “disgusting” (for the complete list of items, see Table S2 in the Supplemental Materials). Higher scores corresponded to perceptions of more stigma against PLWH in the community. Cronbach's alpha in the current sample was .93.

#### Relationship quality

In this paper, the *relationship quality variable* is comprised of four components of subjective evaluation of relationship quality, following the theoretical explanations by Fletcher et al. (2000): relationship satisfaction, dyadic trust, intimacy, and commitment. The measures we used included the following: 1) a five‐item subscale of overall *relationship satisfaction* (Rusbult et al., 1998) measured on a Likert scale ranging from 1 (*Do not agree at all*) to 9 (*Agree completely*); 2) an 8‐item scale of *dyadic trust* between partners in relationships (Larzelere & Huston, 1980), including questions on honesty, sincerity, fairness, and reliability measured on a Likert scale ranging from 1 (*Strongly disagree*) to 7 (*Strongly agree*); 3) *intimacy* measured by the Inclusion of the Other in Self Scale (IOS; Aron et al., 1992), which uses a series of pictures of circles of “self” and “other” with increasing degrees of overlap and asks participants to choose the picture that best describes the relationship between themselves (self) and their husband/wife (other), with responses varying from 1 (separate) to 7 (almost complete overlap); and 4) *commitment* measured using an 8‐item scale (Kurdek, 2007) scored based on responses from 1 (*Not at all true*) to 9 (*Extremely true*). The Cronbach's alpha levels for these variables varied from .90 to .97 for women and from .87 to .98 for men. The correlations among these variables ranged between .62–.74 and .56–.77 for women and men, respectively, indicating overlap across these variables. Indeed, the Cronbach alpha levels of the relationship quality variable formed of these four components were .89 for both genders. Consequently, we decided to compute a composite score using these four components (see Pusch et al., 2023, for a similar application). Thus, our relationship quality variable was the average of the z‐scores of relationship satisfaction, dyadic trust, intimacy, and commitment.

*Control variables* used in the analyses included socio‐demographic variables at the time of the assessment, including the women's weeks of pregnancy, women's age, the age difference between the partners, women's number of living children, the couple's relationship duration, women's number of years living with HIV, and whether the relationship was a polygamous or non‐polygamous marriage.

### Analysis strategy

We employed a moderation extension of the Actor‐Partner Interdependence Model (APIM; Kenny et al., 2006), using the full information maximum likelihood estimation method with robust standard errors (i.e., MLR; Enders & Bandalos, 2001). The APIM included both actor (e.g., the association between participants' perceived stigma and their own mental health) and partner effects (e.g., the association between participants' perceived stigma and their partner's mental health) of the standardized versions of the HIV‐related stigma and relationship quality variables. Furthermore, the interactions between the participants' HIV‐related stigma variables (i.e., negative attitudes about PLWH and perceived community stigma against PLWH) and their relationship quality were placed in the APIMs. Thus, we tested whether the associations of participants' HIV‐related stigma variables with their and their partner's mental health depended on relationship quality. The hypotheses and analysis strategy were preregistered (https://osf.io/zkqby/), and we have explained the justification for a slight deviation from the preregistration (i.e., the decision to use two‐part models in our analysis) below. Analysis was conducted in Mplus Version 8 (Muthén & Muthén, 1998–2017).

**FIGURE 1:**
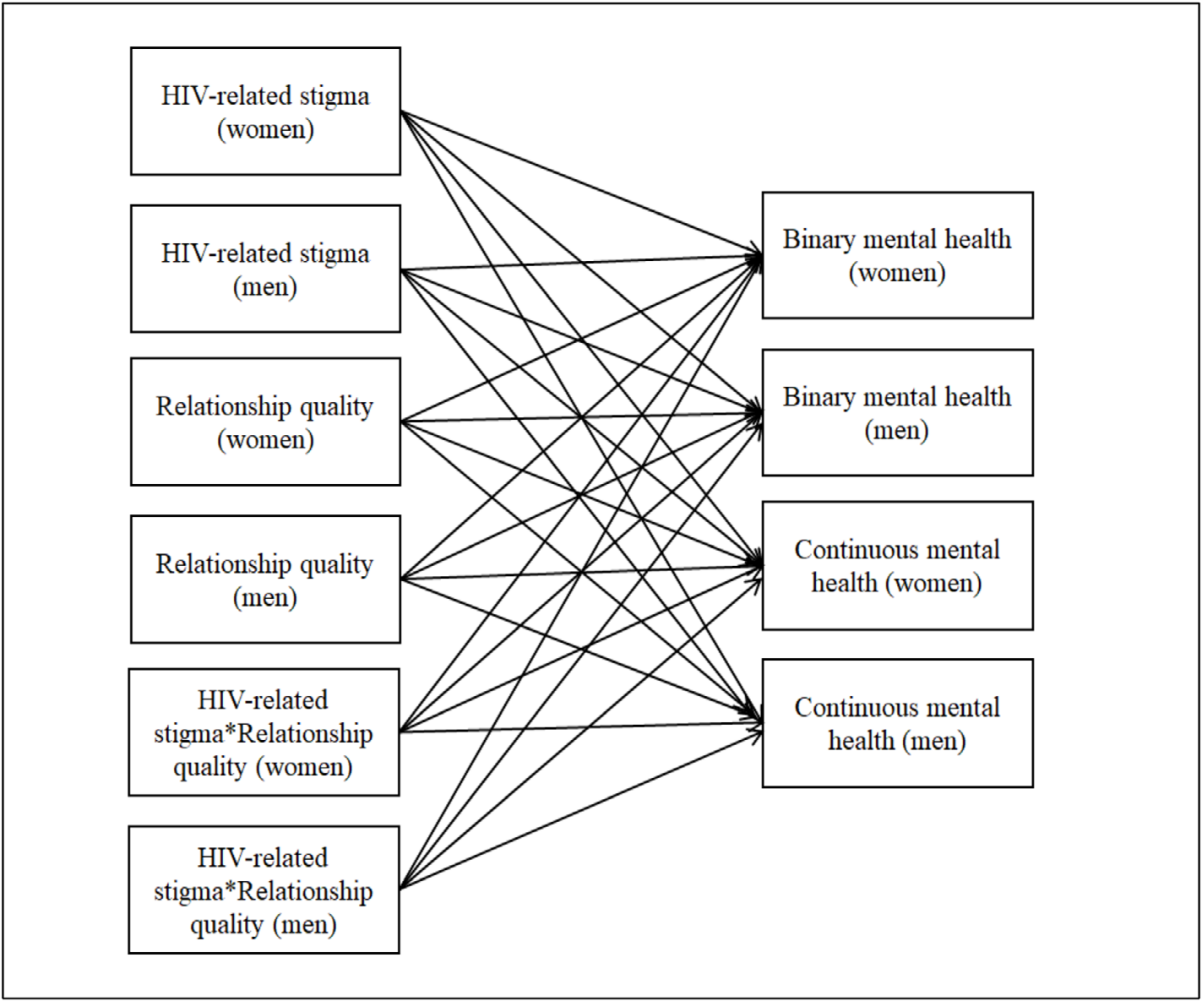
Two‐part actor‐partner interdependence moderation model. *Note.* We used one HIV‐related stigma variable (i.e., negative attitudes about people living with HIV or perceived community stigma against people living with HIV) and one mental health variable (i.e., depression or anxiety symptoms) in each model. This means the above model is used four times, each time including only one stigma and one mental health variable. The binary part of mental health represented zero vs. non‐zero symptoms. The continuous part included non‐zero symptoms. The correlations among the predictor variables and among the residuals are not shown in the figure for the sake of simplicity.

We had four APIMs in total. Each APIM included one of the two HIV‐related stigma variables (i.e., negative attitudes about living with HIV and perceived community stigma against PLWH) and one of the two mental health variables (i.e., depression and anxiety symptoms). That is, the two stigma and two mental health variables did not occur in the same analysis simultaneously. Preliminary examinations of the variables revealed high percentages of participants with zero scores in the mental health variables (i.e., no symptoms of depression or anxiety): 70% of women and 82% of men for depression symptoms and 77% of women and 83% of men for anxiety symptoms. We used two‐part models in our analyses to deal with this highly skewed distribution. That is, we calculated separate estimates for the binary (i.e., zero vs. non‐zero symptoms) and continuous (i.e., non‐zero symptoms) parts of the mental health variables within the same APIM (Figure 1). Given the prevalence of highly skewed data on mental health collected from non‐clinical samples (Gonzalez‐Blanks et al., 2020), this method has been applied in other recent papers examining psychopathological symptoms (e.g., Buyukcan‐Tetik et al., 2024; Zhao et al., 2021). In our case, the binary part results investigated the likelihood of showing any depressive or anxiety symptoms as a function of HIV‐related stigma variables, and the continuous part results examined linear associations between the HIV‐related stigma variables and non‐zero mental health levels.

Regression of both binary and continuous parts of the mental health variables on women's and men's HIV‐related stigma variables, relationship quality, and their interaction terms meant 24 effects in each model (see Figure 1). Following our preregistration, we tested and found that the models with gender equalities across the same effects (e.g., women's actor effect = men's actor effect) did not fit the data worse than the models without constraints (see the Supplemental Materials Table S3 for chi‐square difference test results). However, considering the theoretical significance of gender to fully understand HIV stigma and the fact that gender also represents the HIV status in our sample (HIV + women coupled with HIV‐ men), we decided to present the results in the models without any constraints across gender. That is, different results could emerge for women and men (see Figure 1).

In subsequent analyses, we checked whether the results held when relationship quality indicators (i.e., relationship satisfaction, dyadic trust, intimacy, commitment) were used in separate models instead of a composite score. The results slightly deviated from those reported below and presented in the Supplemental Materials for transparency purposes (see Supplemental Material Tables S4–S7). However, we preferred to present the results with the composite relationship quality variable in the main text following our preregistration and considering the high reliability of the composite variable. This preference also prevented further complexity in the analysis, which already consists of the binary and continuous parts of two mental health variables, two HIV‐stigma variables, and four relationship quality variables.

## RESULTS

### Descriptive statistics

Descriptive statistics in Table 1 show the averages of HIV‐related stigma and mental health variables for the 491 couples included in the analyses.

**TABLE 1:** Descriptive statistics and correlations among stigma, relationship quality, and mental health indicators.

		1	2	3	4	5	6	7	8	9	10
1	Negative attitudes (w)	‐									
2	Negative attitudes (m)	.40**	‐								
3	Perceived stigma (w)	.55**	.33**	‐							
4	Perceived stigma (m)	.31**	.59**	.49**	‐						
5	Relationship quality (w)	−.24**	−10*	−.30**	−.15**	‐					
6	Relationship quality (m)	−.19**	−.16**	−.17**	−.22**	.51**	‐				
7	Depression symptoms (w)	.21**	.20**	.30**	.26**	−.17**	−.02	‐			
8	Depression symptoms (m)	.18**	.24**	.19**	.23**	−.09*	−.09*	.31**	‐		
9	Anxiety symptoms (w)	.22**	.20**	.32**	.23**	−.18**	−.03	.87**	.27**	‐	
10	Anxiety symptoms (m)	.19**	.23**	.21**	.21**	−.11*	−.14**	.23**	.85**	.21**	‐
	*M*	1.68	1.76	1.74	1.83	0.00	0.00	0.15	0.08	0.12	0.07
	*SD*	0.52	0.51	0.40	0.41	0.86	0.87	0.34	0.23	0.29	0.23

*Note*: Negative attitudes = Negative attitudes about people living with HIV, Perceived stigma = Perceived community stigma against people living with HIV, w = women, m = men. The relationship quality variable is the average score composed of the z‐scores of relationship satisfaction, dyadic trust, intimacy, and commitment, and thus has a mean of zero. The descriptives and correlations of each of these relationship quality variables are presented in the Supplemental Materials (Tables S8–S9). They are not presented here for the sake of simplicity.

* *p* < .05.

** *p = < .01*.

### Correlations

Participants' perceptions of HIV‐related community stigma and negative attitudes about people living with HIV had weak to moderate positive correlations with both their and their partner's depression and anxiety symptoms (Table 1). An individual's higher relationship quality had strong negative correlations with their own depression and anxiety symptoms. Women's higher relationship quality was associated with lower depression and anxiety in their male partner, while men's relationship quality was not significantly associated with depression and anxiety in their female partner. Those who perceived higher HIV‐related community stigma and negative attitudes were more likely to report lower relationship quality. Lastly, partners' reports were positively related to each other across all study variables, except for a nonsignificant association between women's depression symptoms and men's relationship quality, supporting the interdependence between women's and men's experiences.

### Actor‐partner interdependence moderation model (APIM) results

We presented the APIM results using the negative attitudes and perceived stigma in Tables 2 and 3, respectively. The explained variances in depression and anxiety symptoms ranged between 7% and 19% across the models in these tables, showing small‐to‐medium effect sizes (Cohen, 1992). Because 30 out of the 32 interactions (94%) in Tables 2 and 3 were non‐significant, it was not possible to give weight to significant interactions. Thus, we concluded that our hypothesis regarding the interaction effect between stigma and relationship quality on mental health was not supported.

**TABLE 2:** *Two‐part actor‐partner interdependence model results for the joint associations of negative attitudes and relationship quality with mental health symptoms* (N = 491 couples).

	*Symptoms (women, n = 491)*							*Symptoms (men, n = 490)*						
	Binary part				Continuous part			Binary part				Continuous part		
	*b*	*SE*	*Odds* *Ratio*	*p*	*b*	*SE*	*p*	*b*	*SE*	*Odds* *Ratio*	*p*	*b*	*SE*	*p*
*m*													
NA (women)	**0.29**	**0.11**	**1.34**	.**010**	0.05	0.06	.477	0.21	0.15	1.24	.153	0.04	0.08	.605
NA (men)	**0.37**	**0.12**	**1.45**	.**002**	0.06	0.07	.349	**0.54**	**0.16**	**1.72**	.**001**	**0.14**	**0.07**	.**038**
RQ (women)	**−0.32**	**0.13**	**0.73**	.**016**	**−0.17**	**0.06**	.**006**	0.04	0.15	1.05	.773	**−0.22**	**0.09**	.**011**
RQ (men)	**0.33**	**0.13**	**1.39**	.**010**	0.01	0.07	.922	−0.02	0.19	0.98	.915	0.01	0.07	.913
NA (women) X RQ (women)	−0.02	0.11	0.98	.825	−0.03	0.05	.632	−0.20	0.13	0.82	.129	**0.13**	**0.06**	.**021**
NA (men) X RQ (men)	0.11	0.12	1.11	.352	0.04	0.07	.530	0.08	0.16	1.09	.611	−0.02	0.06	.696
*Anxiety symptoms*														
NA (women)	0.19	0.12	1.20	.133	0.10	0.07	.150	**0.34**	**0.14**	**1.41**	.**016**	0.04	0.09	.678
NA (men)	**0.46**	**0.13**	**1.59**	.**001**	0.05	0.07	.458	**0.46**	**0.15**	**1.59**	.**002**	**0.18**	**0.09**	.**033**
RQ (women)	**−0.36**	**0.14**	**0.70**	.**011**	**−0.17**	**0.07**	.**016**	−0.16	0.16	0.85	.313	−0.05	0.10	.605
RQ (men)	**0.29**	**0.15**	**1.34**	.**044**	0.11	0.07	.093	−0.08	0.18	0.93	.668	−0.13	0.07	.064
NA (women) X RQ (women)	−0.11	0.11	0.90	.353	−0.08	0.05	.135	−0.02	0.13	0.98	.900	0.09	0.08	.266
NA (men) X RQ (men)	−0.02	0.13	0.98	.891	0.02	0.06	.772	0.03	0.15	1.03	.863	−0.03	0.06	.602

*Note*: NA = Negative attitudes about people living with HIV, RQ = Relationship quality, *SE* = Standard error. Significant results (*p* < .05) are bolded.

**TABLE 3:** Two‐part actor‐partner interdependence model results for the joint associations of perceived stigma and relationship quality with mental health symptoms (N = 491 couples)

	*Symptoms (women, n = 491)*							*Symptoms (men, n = 490)*						
	Binary part				Continuous part			Binary part				Continuous part		
	*b*	*SE*	*Odds* *Ratio*	*p*	*b*	*SE*	*p*	*b*	*SE*	*Odds* *Ratio*	*p*	*b*	*SE*	*p*
*Depression symptoms*														
PS (women)	0.19	0.13	1.21	.140	0.01	0.07	.875	0.16	0.14	1.18	.245	−0.03	0.10	.738
PS (men)	**0.46**	**0.12**	**1.59**	.**000**	**0.14**	**0.06**	.**036**	**0.32**	**0.14**	**1.37**	.**025**	**0.19**	**0.07**	.**010**
RQ (women)	**−0.27**	**0.13**	**0.76**	.**040**	−0.11	0.06	.103	0.07	0.15	1.08	.627	−0.13	0.08	.127
RQ (men)	**0.34**	**0.13**	**1.40**	.**008**	−0.02	0.08	.837	−0.05	0.18	0.95	.767	−0.01	0.07	.859
PS (women) X RQ (women)	−0.07	0.10	0.94	.508	**−0.11**	**0.04**	.**006**	−0.18	0.11	0.84	.107	−0.00	0.07	.945
PS (men) X RQ (men)	0.08	0.11	1.09	.473	0.07	0.07	.308	0.01	0.15	1.01	.941	−0.04	0.07	.674
*Anxiety symptoms*														
PS (women)	0.08	0.15	1.08	.605	**0.17**	**0.08**	.**031**	0.26	0.14	1.30	.061	0.15	0.08	.068
PS (men)	**0.59**	**0.13**	**1.80**	.**000**	0.01	0.08	.875	**0.32**	**0.13**	**1.37**	.**017**	0.10	0.09	.272
RQ (women)	**−0.33**	**0.15**	**0.72**	.**024**	−0.11	0.08	.145	−0.13	0.16	0.88	.423	−0.04	0.10	.736
RQ (men)	**0.31**	**0.14**	**1.37**	.**024**	0.08	0.08	.332	−0.08	0.17	0.92	.639	**−0.18**	**0.08**	.**018**
PS (women) X RQ (women)	−0.16	0.11	0.86	.140	−0.06	0.05	.228	−0.01	0.11	0.99	.897	0.07	0.06	.254
PS (men) X RQ (men)	−0.02	0.12	0.98	.840	0.03	0.08	.699	−0.11	0.15	0.90	.477	0.12	0.07	.098

*Note*: PS = Perceived community stigma against people living with HIV, RQ = Relationship quality, *SE* = Standard error. Significant results (*p* < .05) are bolded.

However, we found evidence for the independent main effects of HIV‐related stigma (perceived community stigma and negative attitudes) and relationship quality on mental health symptoms of both partners. First, both negative attitudes and perceived stigma had adverse actor and partner effects on the binary and continuous parts of depression and anxiety symptoms in both men and women. For example, as shown in Table 2, men's negative attitudes about PLWH were positively associated with their (b = 0.54, p = <.01, odds ratio = 1.72) and their female partner's (b = 0.37, p = <.01, odds ratio = 1.45) likelihood of showing symptoms of depression as well as their own levels of depressive symptoms (b = 0.14, p < .05). We should note that the independent main effects of HIV‐related stigma did not occur in all examinations, but around half of the effects were significant (see Tables 2 and 3).

Regarding the main effect of relationship quality on mental health, we again found some evidence. To illustrate, as shown in Table 2, relationship quality had a negative linear association with anxiety symptoms (i.e., actor effects) in women (b = −0.17, p < .05). Unexpectedly, women's and men's partner effects were in opposite directions. For example, while women's perception of good relationship quality was protective against and thus negatively associated with men's depressive symptoms, b = −0.22, p < .05; Table 2), men's perception of good relationship quality was positively or not significantly associated with women's depression and anxiety measures (e.g., likelihood of showing depression symptoms, b = 0.33, p < .01, odds ratio = 1.39; Table 2). Overall, nearly half of the actor and partner effects of relationship quality were significant (see Tables 2, 3).

### Sensitivity analyses

We also examined whether any demographic variable should be added to the analyses as a control variable. To test this possibility, we examined the correlations between both partners' mental health variables (depression and anxiety symptoms) and the woman's weeks of pregnancy, their and their partner's ages, the age difference between the partners, both partners' number of living children, relationship duration, number of years living with HIV, and women's reports about whether their partner has another wife or not (see our preregistration). None was significant except for one association (i.e., the negative correlation between men's age and women's anxiety symptoms) (see Supplemental Materials Table S10 for the complete list of correlations). The results did not change when men's age was added to the models of women's anxiety symptoms. We also explored the results depending on the disclosure status of women (i.e., whether women had shared their HIV + status with their partner) using multigroup analysis and presented them in the Supplemental Materials Tables S11–S12.

Considering the non‐significant difference between models with and without constraints across genders (see the Analysis Strategy), we also present the results with equality constraints in the Supplemental Materials (Table S13). Despite some slight differences in the main effects compared to those reported here, only one of the 16 interaction effects between HIV‐related stigma and relationship quality was significant, again showing a lack of support for our hypothesis of the buffering role of relationship quality.

## DISCUSSION

This study examined the joint effects of HIV‐related stigma and couple relationship quality on mental health among sero‐discordant married couples, where the women were pregnant and living with HIV and their partners were not living with HIV. The study was conducted in a high HIV prevalence region of Kenya. We employed an Actor‐Partner Interdependence Model as the analytical method to examine the effects of HIV‐related stigma on the mental health of these couples, potentially moderated by relationship quality. We found that HIV‐related stigma and relationship quality had independent associations with the mental health of both pregnant women living with HIV and their partners.

We hypothesized that good relationship quality would buffer the adverse effects of HIV‐related stigma on the mental health of sero‐discordant couples. However, our study did not find evidence for such a buffering role of relationship quality. One reason for the lack of an interaction effect in our data could be selection bias in our sample of couples with relatively high relationship quality and good mental health due to the study's inclusion criteria of being in a stable couple relationship. Supporting that speculation, Park et al. (2020) found that participants of dyadic studies in relationship science are more committed on average than individual participants. Another possibility, considering the many individual, relational, and socioecological factors influential on the consequences of HIV stigma, is perhaps that relationship quality acts as a barrier against the harm of stigma depending on the absence of other potent stressors (e.g., unintended pregnancy) or the presence of additional support mechanisms (e.g., from other family members).

We should also note that our focus on relationship quality was limited to each partner's separate report, despite the tests of actor and partner effects. However, the mutuality of relationship quality, the partners' perceptions of each other's relationship quality, or the desired relationship quality from the partner may also matter. Perhaps relationship quality's buffering role occurs when both partners have high levels of relationship quality, or one perceives the partner as having high levels of relationship quality, or one's perception of the partner's relationship quality matches one's desire (cf., Donato et al., 2015; Pollmann & Finkenauer, 2009; Pusch et al., 2023). All these scenarios may elicit feelings of safety and communal coping in the partners and thus add to their mental and physical health (Lewis et al., 2006). Nevertheless, such examinations were not feasible in our study, considering the lack of some of these assessments and the already complicated two‐part dyadic moderation models. However, despite the lack of interaction, we found independent main effects of high levels of HIV‐related stigma and low relationship quality on partners' mental health, as elaborated below.

Our results support the notion that HIV‐related stigma is destructive to the mental health of pregnant women living with HIV and of their partners not living with HIV. Similar findings have been reported previously, with studies suggesting the detrimental impact of HIV stigma on women's mental health (Orza et al., 2015; Waldron et al., 2021). Poor mental health as a result of HIV‐related stigma has also been linked to poor physical health in prior research (Mukerji et al., 2023; Norcini‐Pala et al., 2025). However, our findings suggest that HIV‐related stigma not only harms the mental health of women living with HIV but that the detrimental impact of stigma also extends to the mental health of their male partners not living with HIV. This finding aligns with similar findings from earlier studies suggesting the interdependence of partners' experiences as a function of HIV‐related stigma. For example, findings suggest that stigma beliefs in Chinese sero‐discordant couples and spouses' perceived discrimination are associated with considerable distress to both partners of a dyad (Yu et al., 2016) and that resilience is lower when HIV stigma is high (Huang et al., 2018). Such findings underscore the importance of developing couples‐based interventions that incorporate the mental health of both partners in a discordant relationship rather than focusing only on the partner living with HIV.

We also found that good relationship quality can independently be protective of mental health in HIV sero‐discordant couples. This finding means that couple relationship counseling and support for both partners could help improve the mental health of couples affected by HIV. As poor mental health related to HIV stigma has been found to lead to poor HIV‐related health behaviors, e.g., ART adherence (Turan, Crockett, et al., 2019; Turan, Rice, et al., 2019), our findings have implications for improving HIV‐related health. For example, a recent dyadic study from Malawi suggested that ART non‐adherence is associated with high levels of anticipated stigma when general support from partners and sexual satisfaction were low (Gutin et al., 2023). However, this effect of stigma on ART non‐adherence was mitigated for those in supportive and sexually fulfilling relationships. These findings held for both discordant and concordant partners (Gutin et al., 2023). Similarly, a qualitative study with 40 couples from Kenya suggested that the couples who were more strongly invested in their relationship reported more HIV health‐enhancing behaviors such as status disclosure to partners, regular clinic visits, and better ART adherence (Rogers et al., 2016). Interventions that target couples affected by HIV and include couple relationship strengthening components have promising results for HIV behaviors and health outcomes in recent studies in the African region (Conroy et al., 2024; Darbes et al., 2019; Turan et al., 2018).

Interestingly, we found that some of the partner effects of women's and men's relationship quality were in opposite directions. For example, women's report of good relationship quality was protective against and thus negatively associated with men's depressive symptoms. In contrast, men's report of good relationship quality was positively associated with women's likelihood of showing depression symptoms. Although this unexpected negative association between the partner's perception of relationship quality on women's mental health should be interpreted cautiously until replicated, explanations from two perspectives on this result are plausible: gender differences and differential HIV statuses of partners. First, due to the traditional gender roles, women may blame themselves for adversely impacting their partner's social status and being a source of shame in society (Kohler et al., 2014), especially when their partner is committed to the relationship. Second, seeing the partner's investment in the relationship might intensify the possible guilt of the partner living with HIV for the likelihood of transferring the virus to their unborn baby. Thus, future studies should test the possible combined mechanisms of gender and HIV status in this result.

This study has several strengths, including a large sample of sero‐discordant couples (*N* = 491 couples) and detailed measurement of couple relationship factors, stigma, and mental health simultaneously from both partners of the couple. Regarding the limitations, first, our study included a research design where the sero‐discordant couples were composed of only women living with HIV and men not living with HIV. This design meant that the mental health effect of stigma and the buffering role of relationship quality could not be captured for couple dyads where the man was living with HIV and the woman was not living with HIV. Additionally, men's HIV‐negative status was based on men's self‐report and may not have always been accurate. We invite future researchers to investigate our research questions in couples where both partners are living with HIV. In addition, only stable couples who were willing to take part in a randomized trial of interventions addressing HIV prevention around the time of pregnancy, together as a couple, were included. This meant that couples who did not want to participate in the trial, possibly due to poor relationship quality or fears of stigma, may have been excluded. In addition, the average depression and anxiety symptoms were low in the sample. The zero‐inflated nature of our data, and thus the two‐part analysis we conducted, also prevented us from evaluating the model's fit. Future studies in samples with higher levels of mental health challenges are awaited to address this limitation.

Another limitation is the fact that this study only assessed HIV‐related stigma, although we are aware that many people experience intersecting forms of stigma due to other aspects of their health, behaviors, or identities. Given that intersectional stigma likely better reflects the real‐life complexity and elicits a greater psychological burden than any isolated stigma (Turan, Elafros, et al., 2019), research on individual and couple dynamics related to intersectional stigma is needed. We also note that this study was carried out in counties in Kenya with high HIV prevalence, and results may not be generalizable to areas with lower prevalence of HIV, where HIV‐related stigma may be experienced differently. Finally, this was a cross‐sectional observational study, and any claims about causality or the direction of effects should be made with caution.

## CONCLUSION

HIV‐related stigma and poor relationship quality appear to have detrimental effects on the mental health of pregnant women living with HIV and their partners. Future research should examine these relationships in longitudinal studies to better understand the causality and directionality of effects. Different types of couples should also be included to analyze gender effects. Couples should not only be empowered to resist and reduce HIV‐related stigma, but interventions that incorporate building positive couple relationship skills can help improve the mental health of couples affected by HIV.

## AUTHOR CONTRIBUTIONS

**Asuman Buyukcan‐Tetik**: Conceptualization, Methodology, Formal Analysis, Writing‐Original Draft, Writing‐Review and Editing, Visualization. **Turan Deniz Ergun:** Formal Analysis, Visualization, Writing‐Review and Editing. **Bulent Turan:** Conceptualization, Methodology, Writing‐Review and Editing. **Reshmi Mukerji:** Writing‐Original Draft, Writing‐Review and Editing. **Kevin Owuor:** Data Curation, Writing‐Review and Editing. **Abigail Hatcher:** Conceptualization, Writing‐Review and Editing. **Elizabeth A. Bukusi:** Conceptualization, Supervision, Writing‐Review and Editing. **Zachary Kwena:** Conceptualization, Supervision, Writing‐Review and Editing. **Anna Helova:** Project Administration, Writing‐Review and Editing. **Evelyne Owengah:** Investigation, Supervision, Writing‐Review and Editing. **Lynae Darbes:** Conceptualization, Methodology, Writing‐Review and Editing, Funding Acquisition. **Janet M. Turan:** Conceptualization, Methodology, Writing‐Original Draft, Writing‐Review and Editing, Funding Acquisition.

## CONFLICT OF INTEREST STATEMENT

The authors declare that they have no known competing financial interests or personal relationships that could have appeared to influence the work reported in this article.

## ETHICS STATEMENT

The study was approved by the ethics committees of the University of Alabama at Birmingham and the Kenya Medical Research Institute, and is registered with clinicaltrials.gov (#NCT03547739). All participants provided individual written informed consent.

## Supporting information


**Tables S1‐13.** Excel file for the supplemental information.

## Data Availability

De‐identified data used in this study are available from Janet M. Turan upon reasonable request, subject to a data use agreement. Contact jmturan@uab.edu for access requests. Access is restricted due to ethical considerations and privacy concerns for sensitive participant information.
